# Differential regulation of gene products in newly synthesized *Brassica napus *allotetraploids is not related to protein function nor subcellular localization

**DOI:** 10.1186/1471-2164-8-56

**Published:** 2007-02-21

**Authors:** Warren Albertin, Karine Alix, Thierry Balliau, Philippe Brabant, Marlène Davanture, Christian Malosse, Benoît Valot, Hervé Thiellement

**Affiliations:** 1 UMR de Génétique Végétale, INRA/CNRS/UPSud/INA P-G, La ferme du Moulon, 91190 Gif-sur-Yvette, France; 2 Plate-forme de Protéomique, La ferme du Moulon, 91190 Gif-sur-Yvette, France; 3 Plate-forme de Protéomique de Versailles, INRA, 78026 Versailles, France

## Abstract

**Background:**

Allopolyploidy is a preeminent process in plant evolution that results from the merger of distinct genomes in a common nucleus *via *inter-specific hybridization. Allopolyploid formation is usually related to genome-wide structural and functional changes though the underlying mechanisms operating during this "genomic shock" still remain poorly known. The aim of the present study was to investigate the modifications occurring at the proteomic level following an allopolyploidization event and to determine whether these changes are related to functional properties of the proteins. In a previous report, we applied comparative proteomics to synthetic amphiploids of *Brassica napus *and to its diploid progenitors *B. rapa *and *B. oleracea*. Although several hundred polypeptides displayed additivity (*i.e. *mid-parent values) in the amphiploids, many of them showed non-additivity. Here, we report the *in silico *functional characterization of the "non-additive" proteins (the ones with a non-additive pattern of regulation) in synthetic *B. napus*.

**Results:**

The complete set of non-additive proteins (335 in the stem and 205 in the root), as well as a subset of additive polypeptides (200 *per *organ), was identified by mass spectrometry. Several protein isoforms were found, and most of them (~55%) displayed "different" or "opposite" patterns of regulation in the amphiploids, *i.e. *isoforms of the same protein showing both up-regulation and down-regulation in the synthetic *B. napus *compared to the mid-parent value. Components of protein complexes were identified of which ~50% also displayed "different" or "opposite" patterns of regulation in the allotetraploids. *In silico *functional categorization of the identified proteins was carried out, and showed that neither functional category nor metabolic pathway were systematically affected by non-additivity in the synthetic amphiploids. In addition, no subcellular compartment was found to be over- or under-represented among the proteins displaying non-additive values in the allopolyploids.

**Conclusion:**

Protein identification showed that functionally related polypeptides (isoforms and complex subunits) could be differentially regulated in synthetic *B. napus *in comparison to its diploid progenitors while such proteins are usually expected to display co-regulation. The genetic redundancy within an allopolyploid could explain why functionally related proteins could display imbalanced levels of expression. No functional category, no metabolic pathway and no subcellular localization was found to be over- or under-represented within non-additive polypeptides, suggesting that the differential regulation of gene products was not related to functional properties of the proteins. Thus, at the protein level, there is no evidence for the "genomic shock" expected in neo-polyploids and the overall topology of protein networks and metabolic pathways is conserved in synthetic allotetraploids of *B. napus *in comparison to its diploid progenitors *B. rapa *and *B. oleracea*.

## Background

Polyploidization is a widespread phenomenon in eukaryotes, especially in plants where most or all angiosperms are current polyploids or have polyploid origins [1-3]. The last few years have witnessed new insights into the evolutionary and ecological significance of both auto- and allo-polyploidy in plants [4-8], and these attributes have been related to structural and functional genomic changes [9-12]. In particular, the analyses of synthetic allopolyploids have revealed extensive modifications in gene expression. According to the "additivity" hypothesis, newly-synthesized allopolyploids are supposed to display mid-parent expression patterns. Many exceptions were found in synthetic allopolyploids (*e.g. Arabidopsis *[11], *Senecio *[13], *Brassica *[14], *Triticum *[15], and *Gossypium *neo-polyploids [16]), suggesting that the differential regulation of gene expression is a common feature of plant allopolyploids. Although the phenomenon of non-additive expression in inter-specific hybrids and allopolyploids is now well described, the underlying mechanisms are still poorly understood. Several approaches may be developed to decipher the mechanisms of gene regulation in polyploids. The investigation of candidate causal processes, such as epigenetic mechanisms or RNA inactivation [17,18], may give valuable information. Another main research strategy is based on the characterization of the whole set of genes displaying non-additive expression to understand if gene regulation is related to functional or structural properties of the corresponding proteins. This question was addressed to some extent in synthetic allotetraploids of *Arabidopsis suecica*, at the transcript level using microarrays to identify the genes displaying expression divergence from the mid-parent value [11]. However, since mRNA abundances and protein amounts are poorly related [19], it is difficult to infer over- or under-representation of some protein functions and/or metabolic pathways from transcriptomic data alone. Thus, proteomic approaches constitute an alternative to determine if some functional categories or metabolic networks are over- or under-represented, or if the subcellular localization of the proteins is modified in an allopolyploid context.

In a previous work, we applied comparative proteomics to newly synthesized oilseed rape: *Brassica napus *allotetraploids were synthesized *de novo *from inter-specific hybridization between *B. oleracea *and *B. rapa*, followed by colchicine-induced chromosome doubling [14]. The additivity hypothesis (predicting a mid-parent proteome for the amphiploids) was tested in two distinct organs, the stem and root (see [14] for details). Most spots (519 in stem and 583 in root) displayed a mid-parent value in synthetic *B. napus *("additive" polypeptides), while numerous others (335 in stem, 205 in root) displayed non-additive amounts [14]. Only a subset (~150) of these non-additive polypeptides were identified by mass spectrometry and submitted to *in silico *analyses [14]. In the present study, it is the whole set of non-additive proteins in synthetic *B. napus *(335 in the stem and 205 in the root, that is to say almost four hundreds supplementary "non-additive" polypeptides) that has been identified by mass spectrometry, as well as an additional two hundred "additive" proteins *per *organ that constitute the "control" group. Only such numerous data could allow an accurate and relevant analysis of the functional properties of the proteins in the synthetic allopolyploids. Thus, we report the thorough *in silico *analysis of protein functions, isoforms, complex subunits and subcellular localizations of the additive and non-additive proteins in synthetic amphiploids of *B. napus*.

## Results

Comparative proteomics was applied to the stem and the root of synthetic *B. napus *and its diploid progenitors *B. rapa *and *B. oleracea*. The whole set of spots displaying non-additivity (*i.e. *spots whose abundance differed from the mid-parent value in the amphiploids) were excised (335 in stem, 205 in root, Table 1), as well as a subset of "additive" polypeptides randomly cut out (200 in stem, 200 in root). MALDI-TOF or nanoHPLC-MS/MS mass spectrometry allowed the identification of ~70% of the excised spots in both organs (see also complete protein identification results in Additional file 1).

### Protein isoforms can be differentially regulated

**Figure 1:**
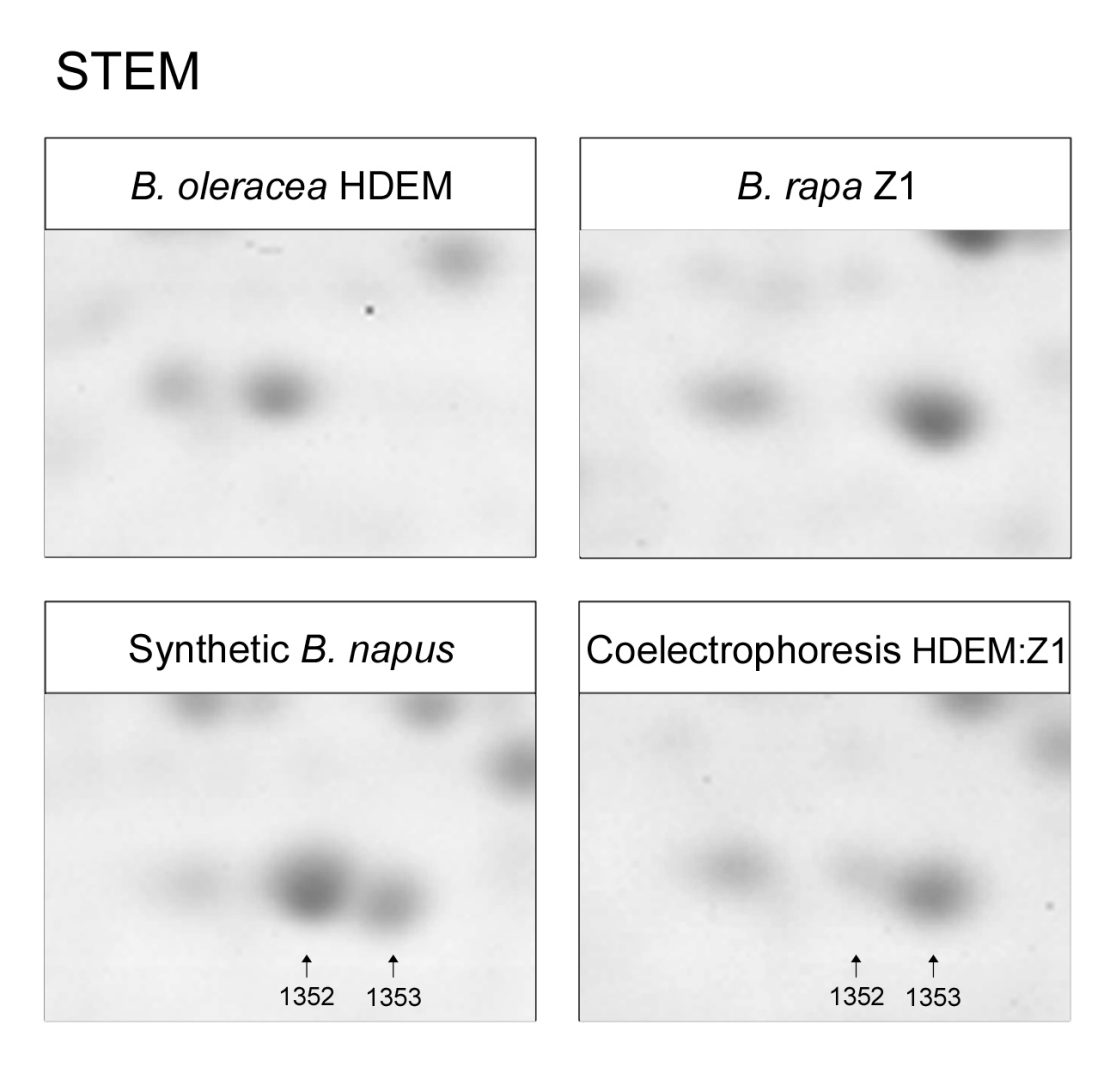
**Opposite patterns of regulation between probable homeoallelic gene products**. 2-DE gels of *B. oleracea *HDEM, *B. rapa *Z1, and synthetic *B. napus *stem proteomes. A coelectrophoresis of HDEM:Z1 stem extracts was added (mid-parent pattern). Spots #1352 and #1353 were HDEM- and Z1-specific respectively, and were both identified as putative fructokinases (At1g06020). These probable homeoallelic gene products displayed ''opposite'' patterns of regulation in synthetic *Brassica napus *in comparison to the mid-parent pattern: spot #1352 was up-regulated while spot #1353 was down-regulated.

Protein identification from two-dimensional electrophoresis gels usually allows the detection of several isoforms [20,21]. Protein isoforms may be *i- *the results of post-translational modifications (PTMs) of the same gene product, *ii- *gene products of paralogous genes, *iii- *gene products of homeologous genes (*i.e. *orthologous genes from the two distinct parental species merged in a common nucleus *via *hybridization) and *iv- *a combination of these different origins. Since the expression of isoforms may be controlled by partially shared or conserved regulatory pathways, it can be hypothesized that they should display an identical regulation within the same organ (for example, a proportional increase in their quantity). This hypothesis was tested in synthetic *Brassica napus*: the identification of spots (displaying additivity and non-additivity) allowed the detection of groups of isoforms in both organs: 104 groups of isoforms in the stem and 82 in the root (Table 2). Among these groups, 42 to 48% comprised spots that all displayed an identical regulation within one organ (all isoforms up-regulated, or all isoforms down-regulated in the synthetic amphiploids compared to the mid parent value), in accordance with the above hypothesis. Interestingly, 15 to 24% of the groups included isoforms with "opposite" regulation patterns, resulting in partial or complete compensation: for example, in the stem, spots #1352 and #1353 were respectively *B. oleracea*- and *B. rapa*-specific and were both identified as putative fructokinases (Figure 1). These proteins were likely encoded by homeologous genes, and both displayed non-additive patterns in synthetic *Brassica napus*: spot #1352 was up-regulated compared to the mid parent value, and such an increase was partially balanced by the down-regulation of spot #1353. The differential regulation of homeologous gene products in allopolyploids appeared as a relatively frequent mechanism, since 7 out of 14 of the putative pairs of homeologous proteins displayed contrasting patterns of regulation.

**Figure 2:**
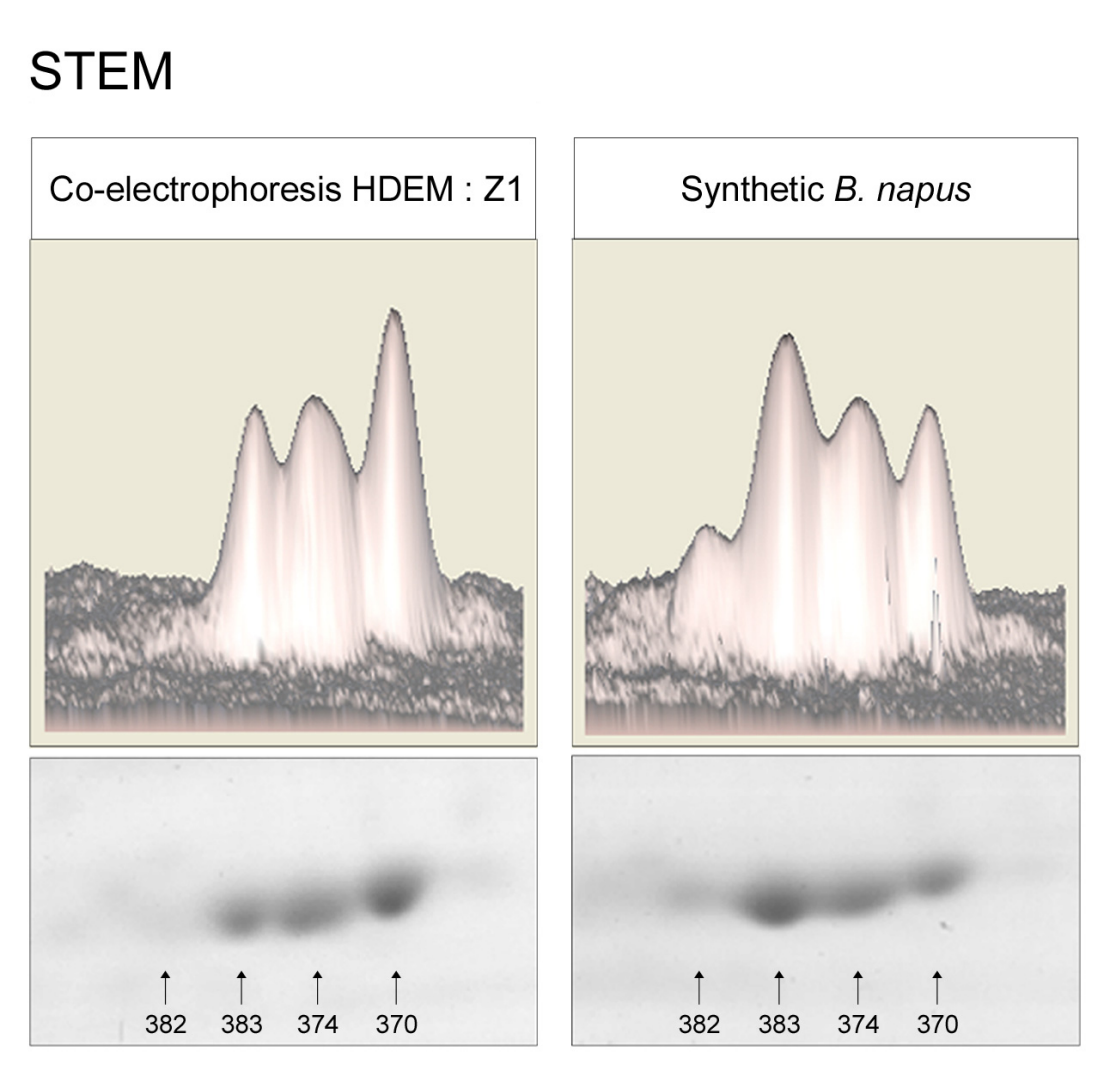
**Opposite patterns of regulation between probable post-translationally modified isoforms**. Two-dimensional and three-dimensional view of 2-DE stem gels. Spots #382, #383, #374 and #370 were identified by mass spectrometry as isoforms of heat shock protein 70-7 (At5g49910) and were probable post-translational modifications of the same gene product. Spot #374 displayed additivity in synthetic *B. napus*, while spot #370 was down-regulated and spots #382 and #383 were up-regulated compared to the mid parent value.

One of the great advantages of comparative proteomics is the possibility to observe post-translational modifications (PTMs) of the same gene product. Although it is not obvious to demonstrate formally whether multiple isoforms correspond to PTMs or to products of homologous/homeologous genes, proteins with post-translational modifications such as phosphorylation usually appear on the 2-DE gels as horizontal rows of spots, resulting in characteristic "trains of spots". Figure 2 shows a train of four spots (#370, #374, #382 and #383) for which analysis by mass spectrometry led to the identification of one single accession, namely an Hsp70-like protein. Moreover, previous studies have described post-translational modifications of HSP 70-related proteins [22,23] so that the four detected spots most likely corresponded to different PTMs of HSP 70. Quantitative proteomic analysis revealed that spot #374 was additive in the synthetic *B. napus*, while isoform #370 was less abundant in amphiploids than expected under the additivity hypothesis whereas spots #382 and #383 were more abundant. Other examples of trains of spots with contrasting patterns of regulation were detected (26/44 for both organs), suggesting that the differential regulation of PTMs was not a rare event in the synthetic amphiploids of *B. napus*.

In order to know whether the distribution of the isoforms was similar between polypeptides displaying additivity and non-additivity, we counted the number of isoforms (Table 3). In both organs, significantly more isoforms were found among non-additive spots than among spots displaying additivity (χ^2 ^tests, *P-values *= 3.76^e-6 ^and 1.42^e-10 ^in the stem and the root respectively, both inferior to the *p-value *= 0.05 limit). Such a difference might reflect the experimental bias due to the differential sampling of additive and non-additive spots. Since we analyzed by mass spectrometry all the spots with non-additive values, we detected all the groups within which at least two isoforms displayed non-additive patterns. On the other hand, we identified only a random subset of the additive spots (two hundred spots per organ while we found 519 and 583 additive spots in the stem and root respectively), so we may have underestimated the number of groups of isoforms within the additive group.

### Complex subunits can be differentially regulated

**Figure 3:**
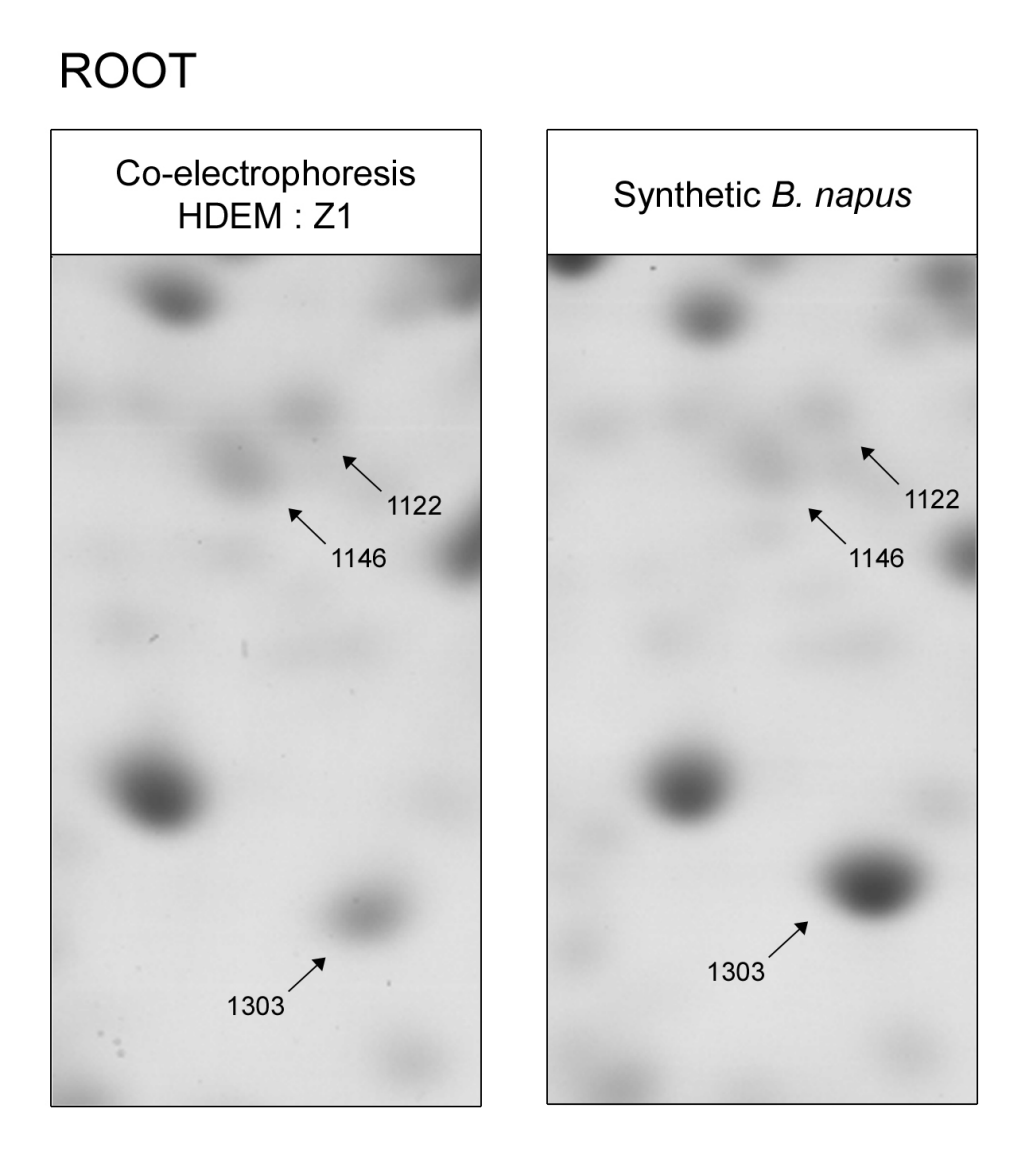
**Opposite patterns of regulation between RPSaA and RPP0A, small and large ribosome subunits**. Spots #1122 and #1146 were identified by mass spectrometry as isoforms of 40S ribosomal protein SA RPSaA (At1g72370). Both spots were down-regulated in synthetic *B. napus *compared to the mid parent value. Spot #1303 was identified as a 60S acidic ribosomal protein P0 RPP0A (At2g40010) and was up-regulated in the amphiploids. RPSaA and RPP0A were respectively small and large ribosome subunits [26].

Most metabolic pathways are driven by protein complexes. Thus, the stoichiometry of the different subunits might be a critical issue for cell processing, and the components of a protein complex are expected to display parallel variations of their respective amounts. These supposed co-regulation and coordinate expressions are known as "the balance hypothesis", and it has been suggested that both under-expression and over-expression (imbalance) of subunits shall be deleterious [24,25]. Hence, the "balance hypothesis" was tested in the synthetic amphiploids of *Brassica napus*: protein complexes with at least two identified distinct subunits were studied. Eleven and nine complexes were found in the stem and root respectively, and the pattern of regulation of their components was investigated (Table 4). For example, analysis of the stem proteome led to the identification of the alpha, beta, delta and gamma subunits of the chloroplastic ATP synthase, and all of these four subunits displayed non-additive up-regulation in *B. napus *synthetic amphiploids compared to the mid parent value. On the contrary, two subunits of the ribosomal complex, the 40S ribosomal protein SA (RPSaA) and the 60S acidic ribosomal protein P0 (RPP0A) [26], were found to be differentially expressed in the root, but with opposite patterns of regulation: two isoforms of RPSaA subunit were down-regulated while RPP0A was up-regulated in the amphiploids (Figure 3). Finally, only 5/11 complexes in stem and 2/9 in root encompassed subunits with identical patterns of regulation, demonstrating that the different subcomponents of a complex can be differentially regulated in neo-synthetized *B. napus*. These results suggest that the balance hypothesis might not be a general rule for the regulation of different complex subunits in synthetic allopolyploids.

### No functional categories are under- or over-represented among proteins displaying non-additivity in synthetic *B. napus*

**Figure 4:**
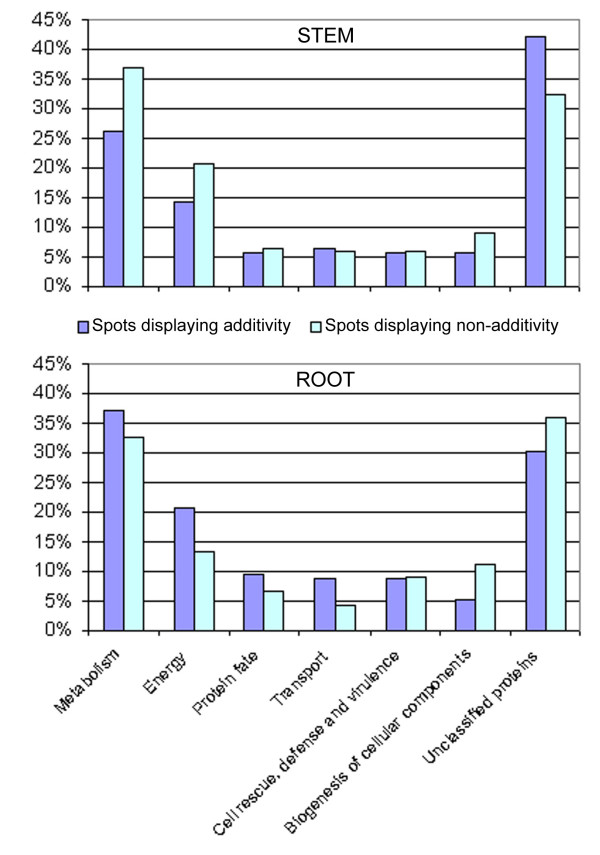
**Functional categorization of the proteins identified in synthetic *Brassica napus***. Each group of isoforms was counted once: in the stem, among the 143 proteins displaying additivity in the amphiploids, 126 were non-redundant and were taken into account. For non-additive spots, 155/234 non-additive spots were non-redundant and analyzed. In the root, 115/140 additive and 89/126 non-additive spots were non-redundant and were taken into account. Functional categorization was made using the MIPS FunCat database [27]. The "transport" category covers cellular transport, transport facilitation and transport routes.

One of the major goals of protein identification of both additive and non-additive spots was to find whether some functions or metabolic pathways were preferentially affected by allopolyploidization. Thus, the functional categorization of the identified polypeptides was carried out using the MIPS FunCat database available on line [27]. Since the proportion of isoforms was significantly imbalanced between additive and non-additive spots, we did not consider the isoforms individually but we counted each group of isoforms as a single protein. Figure 4 shows the distribution of additive and non-additive spots into functional categories. In both organs, the identified proteins were mainly involved in metabolism (26% to 37%) and energy (14% to 21%), which was congruent with previous proteomic data [21,28]. Protein fate, transport, defense and biogenesis of cellular components were other well represented functional categories (5% to 10%), while an important proportion of the identified polypeptides were not assigned to any known function (30% to 42%). No functional category was found over- or under-represented for spots displaying non-additivity compared to "additive" spots (χ^2 ^tests, *p-values *> 0.05), suggesting that differential regulation of gene products in synthetic *Brassica napus *was not related to the function of the proteins.

**Figure 5:**
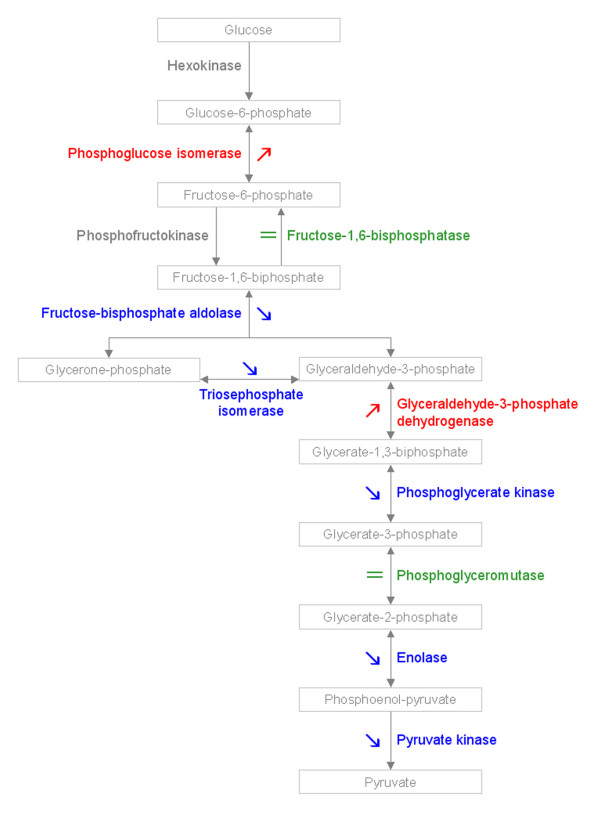
**Patterns of regulation of glycolytic enzymes in synthetic *Brassica napus***. Enzymes up-regulated in synthetic *B. napus *compared to the mid parent value are colored in red, enzymes down-regulated are marked by blue scripts, and enzymes displaying an additive pattern are green in color. The hexokinase and the phosphofructokinase were not identified. The fructose-biphosphate aldolase was globally down-regulated, since four isoforms were found: one being up-regulated, the three others being down-regulated. For the triosephosphate isomerase, two isoforms were found, both of them being down-regulated. For the other enzymes, only one isoform was identified.

In addition, we used the KEGG PATHWAY database [29] to identify the proteins involved in the same metabolic pathways (Table 5). No pathway was particularly altered by differential gene expression, since no proteins involved in the same pathway were found all up-regulated, or down-regulated in the synthetic amphiploids compared to the mid parent value. Moreover, the proteins involved in the same pathway could display different patterns of regulation: for example, most of the glycolytic enzymes were identified in stem proteome (Figure 5). Some of these proteins were up-regulated in the amphiploids (like the phosphoglucoisomerase), others were down-regulated (enolase) or displayed an additive pattern (phosphoglyceromutase). Taken together, these results indicated that neither functional category nor metabolic pathway were particularly targeted by differential regulation of gene expression in the amphiploids.

### Proteins displaying non-additivity in synthetic *B. napus *are not from a peculiar cellular localization

**Figure 6:**
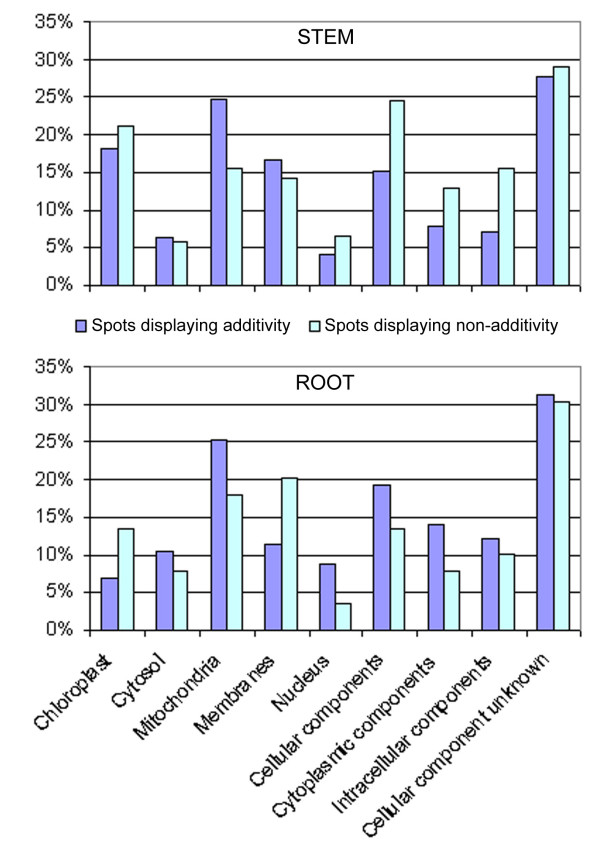
**Cellular localization of the proteins identified in synthetic *Brassica napus***. Each group of isoforms was counted once: in the stem, 126/143 additive and 155/234 non-additive spots were non-redundant and were taken into account. In the root, 115/140 additive and 89/126 non-additive spots were non-redundant and were taken into account. The cellular localization of the proteins was assessed using the Gene Ontology GO annotations [30].

The subcellular localization of the proteins was assessed using the Gene Ontology GO annotations [30]. As for the functional categorization of the proteins, each group of isoforms was taken into account only once in order to correct for the bias between additive and non-additive spots. Figure 6 shows the distribution of the identified proteins within cellular compartments: in both organs, most of the polypeptides were located in the organelles (chloroplast and mitochondria), in accordance with the main functional categories previously found, namely Energy and Metabolism. The fact that ~10% of the root proteins were located in the "chloroplast" actually indicate that these proteins were located in the plastids, and particularly in the amyloplasts, which are very abundant in root tissues [31]. Other cellular localizations, such as cytosol, membrane and nucleus, were well represented in both organs, indicating that comparative proteomics allows the analysis of all the compartments of the cell. The comparison between non-additive and additive spots showed no specific subcellular localization (χ^2 ^tests, *p-values *> 0.05), which suggests that the regulation of gene products in the amphiploids was not related to subcellular localization.

## Discussion

### The differential regulation of isoforms and complex subunits modifies the equilibrium between functionally related proteins in synthetic *Brassica napus*

Protein identification in synthetic amphiploids of *B. napus *allowed the discovery of several isoforms that could display different patterns of regulation. In particular, probable homeoallelic products were found with "opposite" regulation (up- and down-regulation in the synthetic allotetraploids compared to the mid parent value), suggesting that differential expression of homeologous genes could occur. Unequal contributions of homeoalleles to the transcriptome have already been evidenced in synthetic allotetraploids of *Gossypium *[12,32]. Here, we have shown that such a phenomenon was detectable at the proteomic level and was not a rare event. Moreover, our proteomic data suggest that the post-translational modifications of the same gene product could also be differentially regulated in the amphiploids. Differential regulation of PTMs has been described in a few proteomic analyses [33], but no data was available in the context of allopolyploidy. PTMs play a key role in the cell as the same gene product may have different activities, modified turn-overs, distinct subcellular locations, or altered interactions with proteins or nucleic acids depending on its PTMs. Thus, variation of amounts among protein isoforms (PTMs, but also homeoalleles or paralogous genes) may establish a novel metabolic equilibrium in synthetic *Brassica napus *in contrast to its diploid progenitors.

Complex subunits are usually supposed to present co-ordinate expressions [24,25]. Such a statement was supported by some experimental data showing that genes encoding subcomponents of a complex displayed the same pattern of regulation of their expressions [34,35]. In contradiction with this assumption, we have shown that the different subunits of a protein complex could be differentially regulated in newly synthesized *B. napus*. This result may reflect the instability of protein regulation in the very first step of allopolyploidization, and it will be of particular interest to investigate the evolution of such imbalance in the further generations of allotetraploids. Moreover, although the synthetic *B. napus *plants we analyzed were viable and morphologically "normal", neither phenotypic analyses nor relative fitness studies were undertaken. Thus, the imbalanced levels of isoforms and subunit complexes may have an impact on the fitness and/or the phenotype of the neo-allopolyploids that remains to be quantified. One can also suppose that, due to genetic and proteomic redundancies, expression constraints are relaxed within an amphiploid, allowing previously constrained genes to be differentially regulated and to evolve in a different way.

### Differential regulation of gene products is not related to functional properties of the proteins

During the last few years, several synthetic allopolyploids have been investigated at the transcriptional level using various approaches (cDNA-SSCP in synthetic *Gossypium *allotetraploids [12], AFLP- cDNA in synthetic wheat allopolyploids [15], microarray in synthetic *Senecio *allopolyploids [13] and synthetic *Gossypium *allotetraploids [32], etc.). Non-additive transcription in hybrids and allopolyploids is now well documented, yet the genes concerned by non-additive expression are not well characterized. Recently, a transcriptional analysis of *Arabidopsis *allotetraploids showed that genes involved in "hormonal regulation" and in "cell defense and aging" were more susceptible to expression changes [11]. In addition, the ethylene biosynthesis pathway was found globally repressed in two distinct lineages of synthetic *A. suecica *[11]. In this report, we have described a thorough *in silico *functional analysis of proteins in synthetic *B. napus*, and we have shown that neither specific functional category nor metabolic pathway were over- or under-represented among non-additive proteins. This discrepancy between *Arabidopsis *transcriptional results and oilseed rape proteomic data may be explained by the fact that mRNA abundance and protein amounts are poorly related [19]) or by the reference used (we compared the functional categorizations of non-additive proteins to additive ones, while Wang *et al. *compared the distribution among functional categories of non-additive genes to the whole set of 26,000 annotated genes in *Arabidopsis *[11]). Moreover, differential regulation of gene expression may vary from one biological model to an other (*Arabidopsis *vs. *Brassica*).

Finally, this is the first *in silico *analysis of proteins in synthetic allotetraploids. Although *in silico *prediction is not a perfect process and may give erroneous information for some genes considered individually [36], bioinformatic tools are particularly relevant for genome-wide analysis and global consideration of the data. We have shown that non-additive and additive polypeptides could not be differentiated on the basis of their function nor subcellular localization, indicating that the regulation of gene products following allopolyploidization is not related to any functional feature of the proteins.

### Allopolyploidy: a genomic shock but not a proteomic shock?

Polyploidy is often described as a genomic shock [37,38], that may induce stress and defense responses. Our study showed that, in synthetic *B. napus*, the "cell rescue, defense and virulence" category was unchanged in the stem and root. In addition, Wang *et al. *showed that the "defense" class was under-represented when analyzing differential gene transcription in leaves and flower buds of synthetic *Arabidopsis *allotetraploids [11]. These results suggest that neither stress nor defense responses were particularly enhanced following a recent polyploidy event for these two *Brassicaceae *species. Moreover, although stem and root proteomes were extensively modified in synthetic *B. napus*, the patterns were reproducible in independent amphiploid lineages [14] and our data indicated that, globally, the distribution of the proteins into metabolic pathways, functional categories and subcellular localizations was not disturbed. Thus, at the protein level, there is no evidence for a chaos or a large disorder following the merging of two genomes. Instead, a novel order establishes quickly and may evolve in further generations of synthetic *B. napus*. Hence, allopolyploidy can be seen as a mechanism making new from old: new expression patterns from old genomes, and perhaps this feature explains in part why polyploidy is such an undeniable evolutionary success.

### Differential gene expression: what are the underlying mechanisms?

The thorough *in silico *analysis of the non-additive proteins in synthetic *B. napus *indicated that the differential regulation of gene products was not related to functional properties of the proteins. The molecular mechanisms however remain to be investigated, such as the possible implication of transposable elements: more and more data suggest that transposable elements have played a key role in polyploid evolution such as *Gossypium *or *Arabidopsis *[9,39], and that their transcriptional activation could alter the expression of adjacent genes (like in wheat allopolyploids [40]). Epigenetic mechanisms (changes in chromatin structure, histone modification, DNA methylation, etc.) are described in synthetic allopolyploids [41,42] and may be responsible for the modification of gene expression in polyploids [43,44]. Furthermore, RNA interference, action of siRNAs and miRNAs and their implication in gene regulation, are thus good candidates to explain nonadditive gene expression [45]. Finally, allopolyploidy not only induces the merger of homeologous genomes in a common nucleus, but also the juxtaposition of divergent regulatory networks. The interaction and the coordination of such homeologous networks in a newly synthesized allopolyploid may also explain the genome-wide modification of gene expression, as well as the maintenance at a viable level, in successful individual plants, of the equilibrium between the different functions, metabolic pathways and cellular localizations.

## Conclusion

The complete set of proteins displaying non-additivity and a random sample of additive polypeptides were identified by mass spectrometry in synthetic *Brassica napus*. Functionally related proteins, such as isoforms and complex subunits, were detected and some of them displayed different or opposite patterns of regulation. No functional category, no metabolic pathway and no subcellular localization was found over- or under-represented for non-additive polypeptides. These results suggested that the differential regulation of gene products was not related to functional properties of the proteins and that the mechanisms responsible for the regulation of gene expression in synthetic amphiploids (transposon activation ?, epigenetic processes?) were not initiated according to any protein characteristic. To better understand the success of polyploidy, additional work based on transcriptional analysis and study of the post-translational modifications of the non-additive proteins will be necessary. Only such an integrative study would help in understanding the molecular and biochemical mechanisms responsible for the regulation of gene expression which occurs in a polyploid organism.

## Methods

### Mass spectrometry identification of additive and non-additive proteins

In a previous report, we applied comparative proteomics to synthetic *Brassica napus *(four independent lineages) and its diploid progenitors *B. rapa *and *B. oleracea *(two to five plants analyzed *per *genotype) [14]. Our proteomic analysis showed that several spots (519 in stem and 583 in root) displayed a mid-parent value in synthetic *Brassica napus *compared to its diploid progenitors while many others (335 in stem, 205 in root) displayed non-additive amounts [14]. About 150 of these non-additive polypeptides were identified by mass spectrometry and submitted to *in silico *analyses [14]. However, the subset of non-additive polypeptides analyzed was too small to obtain accurate and relevant data on the functional categorization and subcellular localization of the non-additive proteins [14]. Here, the remaining non-additive spots (~260 in stem and ~130 in root) were excised from 2-DE gels and submitted to mass spectrometry, so that the whole set of non-additive proteins in synthetic *B. napus *was identified.

In our previous study, no accurate "control group" was available (no additive spot was identified), so that we used reference proteomic maps of other organisms (*Pisum sativum*, *Medicago truncatula*) [14]. Here, two hundred spots displaying additivity were also cut out for both organs (random excision among the 519 and 583 spots found additive in stem and root [14]). These additive polypeptides were used as controls for subsequent *in silico *analyses. Mass spectrometry analyses were conducted as described in [14].

### Assessing protein isoforms

Distinct spots were considered as isoforms when they met the following criteria: 1 – the corresponding identified protein sequences presented more than 70% identity all along the sequences; 2 – the corresponding proteins had the same function (see below) 3 – the polypeptides were assessed *in silico *to the same cellular compartment (see cellular localization below) and 4 – the spots displayed similar molecular weight on 2-DE gels (to discard degradation or catabolic products).

### *In silico *analyses

Functional categorization and cellular localization of the proteins were conducted using the MIPS FunCat database (Version 2.0) available on line [27] and the TAIR's Gene Ontology (GO) annotations (release of February 2006) [46,47] as described in [14]. For both organs, the main molecular networks were identified using the KEGG PATHWAY database (release of February 2006) [29]. χ^2 ^tests were carried out to test statistical differences between proteins fitting additivity or displaying non-additivity.

## Abbreviations

2-DE: two-dimensional electrophoresis ; PTM: post-translational modification

## Authors' contributions

WA carried out comparative proteomics, analyzed the mass spectra, made in silico analyses and drafted the manuscript. KA and PB participated in the design of the study, the interpretation of the data and helped to draft the manuscript. TB, MD and BV carried out nanoHPLC-MS/MS analyses; CM carried out MALDI-TOF analyses. HT conceived and designed the study, and drafted the manuscript. All authors read and approved the final manuscript.

## Supplementary Material

Additional File 1 **Protein identification in synthetic *Brassica napus***. Patterns: "additive": spot displaying a mid-parent value in the amphiploid compared to its diploid progenitors ; "positive/negative over-dominance": spot displaying a higher/lesser quantification value in the amphiploid compared to the higher/lesser parent ; "HDEM or Z1 dominance": amphiploid spot abundance equaled either HDEM or Z1 value ; "up-regulation" or "down-regulation": spot amount superior or inferior to the mid-parent value ; "Intermediary" pattern: non-additive spot with an abundance ranging between mid-parent and parental values; "progressive": spot abundance increased or decreased progressively with successive amphiploid generations. Click here for file

## Figures and Tables

**Table 1:** Protein identification in synthetic *Brassica napus*.

	Stem				Root			
	
	Spots displaying additivity		Spots displaying non-additivity		Spots displaying additivity		Spots displaying non-additivity	
Number of picked spots	200		335		200		205	
Number of identified spots	143	71.5%	234	69.9%	140	70.0%	126	61.5%

Spots were excised from two-dimensional electrophoresis gels and were identified by mass spectrometry (MALDI-TOF or nanoHPLC-MS/MS).

**Table 2:** Regulation of protein isoforms in synthetic *Brassica napus*.

	Stem		Root	
Group of isoforms showing identical patterns of regulation	44	42.3%	39	47.6%
Group of isoforms showing opposite patterns of regulation	25	24.0%	12	14.6%
Group of isoforms showing different patterns of regulation	35	33.7%	31	37.8%

Total	104		82	

For each organ, protein identification data were used to assess isoforms. Groups of isoforms that comprised spots sharing the same pattern of regulation (*i.e. *all spots up-regulated compared to the mid parent value, all spots down-regulated or all spots displaying additivity) were designated as "Group of isoforms showing identical pattern of regulation". Groups of isoforms that encompassed up-regulated and down-regulated spots were designated as "Group of isoforms showing opposite patterns of regulation". Groups of isoforms that included additive and non-additive spots (amongst which non-additive spots shared identical patterns of regulation, *i.e. *up-regulation or down-regulation) were designated as "Group of isoforms showing different patterns of regulation".

**Table 3:** Protein isoforms amongst spots displaying additivity and non-additivity in *Brassica napus*.

	Stem				Root			
	
	Spots displaying additivity		Spots displaying non-additivity		Spots displaying additivity		Spots displaying non-additivity	
Number of identified spots	143		234		140		126	
Number of protein isoforms	37	25.9%	117	50.0%	46	32.9%	91	72.2%

Significantly more isoforms were found amongst spots displaying non-additivity than amongst spots displaying additivity (χ^2 ^tests, *P-values *< 0.05, *P-value *= 3.76^e-6 ^in the stem and *P-value *= 1.42^e-10 ^in the root).

**Table 4:** Regulation of complex subunits in synthetic *Brassica napus*.

Complexes with at least two identified subunits	Stem	root
Complexes with subunits showing identical regulation patterns	5	2
Complexes with subunits showing opposite regulation patterns	1	3
Complexes with subunits showing differing regulation patterns	5	4

Total	11	9

For each organ, complexes with at least two distinct subunits identified were detected. Those complexes were differentiated on the basis of the regulation patterns of the subunits that could either display an identical pattern of regulation (all subunits up-regulated compared to the mid parent value, or all subunits down-regulated), or show opposite regulation patterns (for example, one subunit up-regulated, the other one down-regulated). Complexes comprising subunits with non-additive patterns and subunits with additive patterns were designated as "Complexes with subunits showing differing regulation patterns".

**Table 5:** The main metabolic pathways identified in stem and root proteomes.

Stem	Spots displaying additivity		Spots displaying non-additivity	
Glycolysis/Gluconeogenesis	6	4.8%	11	7.1%
Citrate cycle (TCA cycle)	7	5.6%	6	3.9%
Pyruvate metabolism	5	4.0%	8	5.2%
Photosynthesis	5	4.0%	9	5.8%
Carbon fixation	5	4.0%	15	9.7%
Proteasome	4	3.2%	8	5.2%
Number of non-redundant proteins	126		155	

Root	Spots displaying additivity		Spots displaying non-additivity	

Glycolysis/Gluconeogenesis	9	7.8%	3	3.4%
Citrate cycle (TCA cycle)	9	7.8%	6	6.7%
Pyruvate metabolism	8	7.0%	4	4.5%
Carbon fixation	7	6.1%	5	5.6%
Number of non-redundant proteins	115		89	

For both organs, the main molecular networks were identified using the KEGG PATHWAY database [29]. No bias was found between spots fitting additivity and spots displaying non-additivity in synthetic *B. napus *(χ^2 ^tests. *P-value *> 0.05).
